# 2-[2-(Trimethyl­silyl)eth­yl]isoindoline-1,3-dione

**DOI:** 10.1107/S1600536809054105

**Published:** 2009-12-24

**Authors:** Ilia A. Guzei, Lara C. Spencer, Uzma I. Zakai

**Affiliations:** a Department of Chemistry, University of Wisconsin–Madison, 1101 University Ave, Madison, Wisconsin 53706, USA

## Abstract

In the course of our studies of silicon-containing anti­cancer compounds, the title compound, C_13_H_17_NO_2_Si, was synthesized. The geometrical parameters including the geometry about the Si atom are typical. The mol­ecules form dimers *via* a weak C—H⋯O inter­action described by the graph set *R*_2_^2^(10). The dimers are assembled in rows stacked in the crystallographic *b*-axis direction *via* π–π inter­actions with a 3.332 (3) Å separation between the rows.

## Related literature

For literature related to drug design see: Bains & Tacke (2003 ▶); Bikzhanova *et al.* (2007 ▶); Franz (2007 ▶); Franz *et al.* (2007 ▶); Gately & West (2007 ▶); Guzei,, Spencer, Zakai & Lynch (2010 ▶); Guzei, Spencer & Zakai (2010 ▶); Lee *et al.* (1993 ▶, 1996 ▶); Sen & Roach (1995 ▶); Showell & Mills (2003 ▶); Tacke & Zilch (1986 ▶); Tsuge *et al.* (1985 ▶); Yoon *et al.* (1991 ▶, 1992 ▶, 1997 ▶). For a description of the Cambridge Structural Database, see: Allen (2002 ▶). Bond distances and angles were confirmed to be typical by a *Mogul* structural check (Bruno *et al.*, 2002 ▶). For graph-set notation, see: Grell *et al.* (1999 ▶).
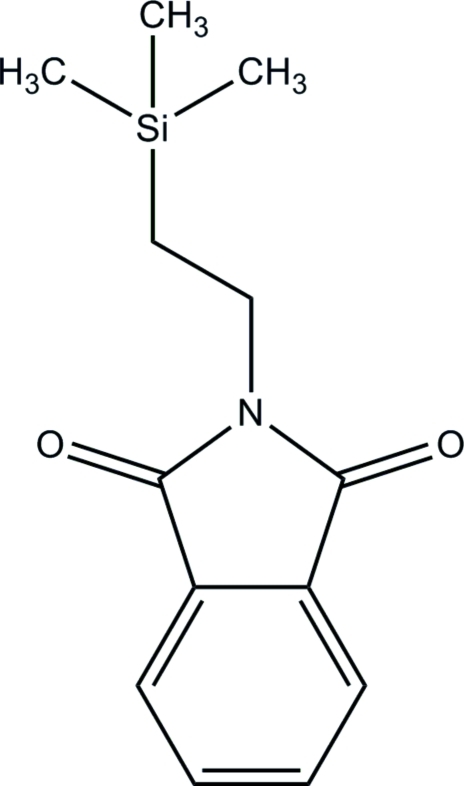


## Experimental

### 

#### Crystal data

C_13_H_17_NO_2_Si
*M* *_r_* = 247.37
Monoclinic, 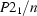

*a* = 11.562 (5) Å
*b* = 6.411 (2) Å
*c* = 19.445 (8) Å
β = 95.176 (14)°
*V* = 1435.5 (10) Å^3^
*Z* = 4
Mo *K*α radiation
μ = 0.16 mm^−1^
*T* = 300 K
0.89 × 0.40 × 0.30 mm

#### Data collection

Bruker SMART X2S diffractometer
Absorption correction: multi-scan (*SADABS*; Bruker, 2009 ▶) *T*_min_ = 0.875, *T*_max_ = 0.955
9164 measured reflections
2701 independent reflections
1750 reflections with *I* > 2σ(*I*)
*R*_int_ = 0.036

#### Refinement

*R*[*F* ^2^ > 2σ(*F* ^2^)] = 0.044
*wR*(*F* ^2^) = 0.151
*S* = 0.99
2701 reflections
157 parameters
H-atom parameters constrained
Δρ_max_ = 0.15 e Å^−3^
Δρ_min_ = −0.16 e Å^−3^

### 

Data collection: *APEX2* and *GIS* (Bruker, 2009 ▶); cell refinement: *SAINT* (Bruker, 2009 ▶); data reduction: *SAINT*; program(s) used to solve structure: *SHELXTL* (Sheldrick, 2008 ▶); program(s) used to refine structure: *SHELXTL*, *OLEX2* (Dolomanov *et al.*, 2009 ▶) and *FCF_filter* (Guzei, 2007 ▶); molecular graphics: *SHELXTL*; software used to prepare material for publication: *SHELXTL*, *modiCIFer* (Guzei, 2007 ▶) and *publCIF* (Westrip, 2010 ▶).

## Supplementary Material

Crystal structure: contains datablocks global, I. DOI: 10.1107/S1600536809054105/zs2025sup1.cif

Structure factors: contains datablocks I. DOI: 10.1107/S1600536809054105/zs2025Isup2.hkl

Additional supplementary materials:  crystallographic information; 3D view; checkCIF report

## Figures and Tables

**Table 1:** Hydrogen-bond geometry (Å, °)

*D*—H⋯*A*	*D*—H	H⋯*A*	*D*⋯*A*	*D*—H⋯*A*
C11—H11⋯O2^i^	0.93	2.57	3.443 (4)	156

Symmetry code: (i) 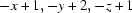.

**Fig. 1.**
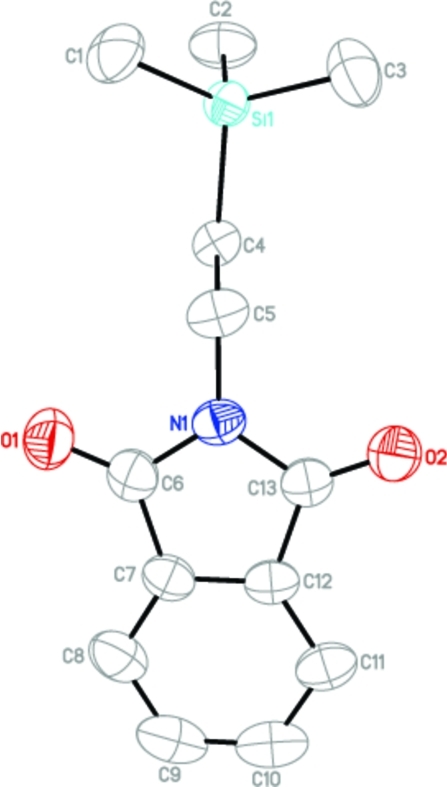
Molecular structure of (I). The thermal ellipsoids are shown at 30% probability level.

## supplementary crystallographic information

### Comment

Sila phthalimides are important intermediates in photochemistry (Lee *et al*., 1993, 1996; Yoon *et al.*, 1997, 1992, 1991) and organic synthesis (Bikzhanova *et al.*, 2007; Tsuge *et al.*, 1985). We have used methods of organosilicon chemistry (Franz, 2007; Franz *et al.*, 2007; Gately & West, 2007; Tacke & Zilch, 1986; Showell & Mills, 2003) to prepare an array of substituted sila amines (Bikzhanova *et al.*, 2007) and to fine-tune the properties of pharmocological drugs (Bains & Tacke, 2003). Sila phthalimides can be obtained from the respective chlorosilanes (Tsuge *et al.*, 1985) or from alcohols by means of the Misunubu reaction (Sen & Roach, 1995) as in the present case. During our research toward silicon-containing anti-cancer drugs the title compound, (I), was isolated and characterized.

The bond distances and angles of (I) are typical as confirmed by the *Mogul* structural check (Bruno *et al.*, 2002), and agree well with those for the related compounds 2-(3-(methyldiphenylsilyl)propyl)isoindoline-1,3-dione (Guzei, Spencer, Zakai & Lynch, 2010) and 2-(((4-methoxyphenyl)dimethylsilyl)methyl)isoindoline-1,3-dione (Guzei, Spencer & Zakai, 2010). Specifically, the average Si—C distances of 1.867 (3) Å for compound (I) are statistically similar to the 1.88 (3) Å average for 83 related compounds in the Cambridge Structural Database (Version 1.11, September 2009 release; Allen, 2002). The Si atom has a distorted tetrahedral geometry with angles ranging from 107.87 (11)° to 110.45 (11) °. The phthalate entity is expectedly planar within 0.0083 Å.

The molecules form dimers *via* a weak C11—H11···O2 interaction with a distance of 3.443 (4) Å and an angle of 155°. The pattern formed can be described in graph set notation as *R*_2_^2^(10) (Grell *et. al*., 1999). The dimers are assembled into rows *via* weak π-π interactions with a distance of 3.366 (5) Å between atoms C13 in separate dimers. The rows are stacked in the crystallographic *b* direction.

### Experimental

The title compound was obtained *via* a Mitsunubu reaction as described by Sen and co-workers (Sen & Roach, 1995). To a pre-dried 100 ml round bottom flask was added 2-(trimethylsilyl)ethanol (319 mg, 2.7 mmol). Additionally, potassium phthalimide (512 mg, 3.48 mmol) and triphenyl phosphine (913, 3.48 mmol) were added to the reaction flask. The flask was sealed with a rubber septum, evacuated, and then filled with an inert atmosphere (nitrogen). Subsequently, 30 ml of freshly distilled THF was added to the round bottom flask. In the dark, the flask was then wrapped with aluminium foil and diisopropyl azodicarboxylate (DIAD) was slowly syringed into the reaction flask. This mixture was allowed to stir at room temperature for four hours. Three ml of water was slowly injected into the reaction mixture, and the given suspension was allowed to stir for a few more minutes. The aluminium foil covering the reaction flask was removed and its contents were poured into an extraction flask. The aqueous phase was extracted 3–5 times with hexane and the resultant organic extracts were dried with MgSO_4_ and filtered. The filtrate was mixed with silica gel and this slurry was dried under reduced pressure. The dry powder was loaded onto a pre-dry packed silica gel column and eluted with a gradient column. The desired material was collected using a 8:2 hexane:ethyl acetate mixture. The compound of interest was dried under reduced pressure and recrystallized from dichloromethane to afford lustrous white needles (0.35 g, 1.41 mmol, 52% yield) for X-ray crystallography. Manipulation of air and moisture sensitive compounds was performed using standard high-vacuum line techniques. All solvents and reagents were obtained from Aldrich. 2-(trimethylsilyl)ethanol was purchased from Gelest. ^1^H NMR spectra were obtained on a Varian Unity 500 spectrometer, ^13^C {H} NMR spectra were obtained on a Varian 500 spectrometer operating at 125 MHz, ^29^Si {H} NMR spectra were obtained on a Varian Unity spectrometer operating at 99 MHz. EI Mass spectra were determined on a Waters (Micromass) AutoSpec mass spectrometer. Melting points were determined on a Mel-Temp Laboratory Device. mp: 48–50°; ^1^H NMR (500 MHz, CDCl_3_) δ 0.04 (s, 9H, Si(Me_3_)_3_), 0.98 (m, 2H, CH_2_), 3.69 (m, 2H, CH_2_), 7.66 (dd, *J*=5.48, 3.01 Hz, 2H, ArH), 7.79 (dd, *J*=5.42, 3.06 Hz, 2H, ArH); ^13^C NMR (125 MHz, CDCl_3_) δ -1.8 (SiMe), 17.0 (CH_2_), 34.4 (CH_2_), 123.0 (CH), 132.3 (CH), 133.7 (CH), 168.2 (CO); ^29^Si NMR (99 MHz, CDCl_3_) 0.01 (Si(Me_3_)_3_); MS (EI^+^) *m/z* (*rel*. intensity %) 247 (*M*^+^, 23), 246 (*M*-1, 100), 232 (50), 204 (75), 160 (26), 130 (55), 91 (49), 73 (39); HRMS (EI^+^): calcd. for C_13_H_17_NO_2_Si (*M*^+^) 247.1024, found (*M*-1)^+^ 246.0945.

### Refinement

All H-atoms were placed in idealized locations and refined as riding with appropriate thermal displacement coefficients *U*_iso_(H) = 1.2 or 1.5 times *U*_eq_(bearing atom). The data were collected at room temperature on a Bruker SMART X2S diffractometer in the automated mode and manually processed thereafter.

### Crystal data

**Table:**

C_13_H_17_NO_2_Si	*F*(000) = 528
*M**_r_* = 247.37	*D*_x_ = 1.145 Mg m^−^^3^
Monoclinic, *P*2_1_/*n*	Mo *K*α radiation, λ = 0.71073 Å
Hall symbol: -P 2yn	Cell parameters from 2755 reflections
*a* = 11.562 (5) Å	θ = 3.4–23.7°
*b* = 6.411 (2) Å	µ = 0.16 mm^−^^1^
*c* = 19.445 (8) Å	*T* = 300 K
β = 95.176 (14)°	Needle, colourless
*V* = 1435.5 (10) Å^3^	0.89 × 0.40 × 0.30 mm
*Z* = 4	

### Data collection

**Table:**

Bruker SMART X2S diffractometer	2701 independent reflections
Radiation source: micro-focus sealed tube	1750 reflections with *I* > 2σ(*I*)
doubly curved silicon crystal	*R*_int_ = 0.036
ω scans	θ_max_ = 25.7°, θ_min_ = 3.4°
Absorption correction: multi-scan (*SADABS*; Bruker, 2009)	*h* = −14→14
*T*_min_ = 0.875, *T*_max_ = 0.955	*k* = −7→7
9164 measured reflections	*l* = −23→23

### Refinement

**Table:**

Refinement on *F*^2^	Primary atom site location: structure-invariant direct methods
Least-squares matrix: full	Secondary atom site location: difference Fourier map
*R*[*F*^2^ > 2σ(*F*^2^)] = 0.044	Hydrogen site location: inferred from neighbouring sites
*wR*(*F*^2^) = 0.151	H-atom parameters constrained
*S* = 0.99	*w* = 1/[σ^2^(*F*_o_^2^) + (0.0922*P*)^2^ + 0.0229*P*] where *P* = (*F*_o_^2^ + 2*F*_c_^2^)/3
2701 reflections	(Δ/σ)_max_ = 0.010
157 parameters	Δρ_max_ = 0.15 e Å^−^^3^
0 restraints	Δρ_min_ = −0.15 e Å^−^^3^

### Special details

**Table:**

Geometry. All e.s.d.'s (except the e.s.d. in the dihedral angle between two l.s. planes) are estimated using the full covariance matrix. The cell e.s.d.'s are taken into account individually in the estimation of e.s.d.'s in distances, angles and torsion angles; correlations between e.s.d.'s in cell parameters are only used when they are defined by crystal symmetry. An approximate (isotropic) treatment of cell e.s.d.'s is used for estimating e.s.d.'s involving l.s. planes.
Refinement. Refinement of *F*^2^ against ALL reflections. The weighted *R*-factor *wR* and goodness of fit *S* are based on *F*^2^, conventional *R*-factors *R* are based on *F*, with *F* set to zero for negative *F*^2^. The threshold expression of *F*^2^ > σ(*F*^2^) is used only for calculating *R*-factors(gt) *etc*. and is not relevant to the choice of reflections for refinement. *R*-factors based on *F*^2^ are statistically about twice as large as those based on *F*, and *R*- factors based on ALL data will be even larger.

### Fractional atomic coordinates and isotropic or equivalent isotropic displacement parameters (Å^2^)

**Table:**

	*x*	*y*	*z*	*U*_iso_*/*U*_eq_	
Si1	0.90115 (5)	0.20211 (9)	0.38252 (3)	0.0610 (3)	
O1	0.75423 (19)	0.2215 (3)	0.61648 (12)	0.1052 (7)	
O2	0.60178 (16)	0.7082 (3)	0.46280 (10)	0.0929 (6)	
N1	0.68492 (15)	0.4340 (3)	0.52646 (10)	0.0686 (5)	
C1	0.8769 (3)	−0.0839 (4)	0.38950 (16)	0.0987 (9)	
H1A	0.9213	−0.1366	0.4299	0.148*	
H1B	0.9009	−0.1522	0.3492	0.148*	
H1C	0.7959	−0.1103	0.3930	0.148*	
C2	1.0592 (2)	0.2585 (5)	0.37947 (16)	0.1010 (9)	
H2A	1.1015	0.2083	0.4209	0.152*	
H2B	1.0705	0.4063	0.3757	0.152*	
H2C	1.0869	0.1900	0.3402	0.152*	
C3	0.8170 (3)	0.3029 (5)	0.30296 (15)	0.1078 (10)	
H3A	0.8418	0.2326	0.2632	0.162*	
H3B	0.8302	0.4500	0.2989	0.162*	
H3C	0.7357	0.2778	0.3058	0.162*	
C4	0.85121 (18)	0.3403 (3)	0.45914 (11)	0.0615 (6)	
H4A	0.8976	0.2924	0.5001	0.074*	
H4B	0.8659	0.4883	0.4543	0.074*	
C5	0.72357 (19)	0.3103 (4)	0.47001 (13)	0.0773 (7)	
H5A	0.6770	0.3469	0.4277	0.093*	
H5B	0.7100	0.1640	0.4792	0.093*	
C6	0.7031 (2)	0.3775 (4)	0.59582 (14)	0.0758 (7)	
C7	0.6491 (2)	0.5451 (4)	0.63477 (13)	0.0724 (6)	
C8	0.6418 (3)	0.5693 (6)	0.70431 (16)	0.0995 (9)	
H8	0.6728	0.4707	0.7359	0.119*	
C9	0.5867 (3)	0.7459 (7)	0.72558 (18)	0.1135 (11)	
H9	0.5795	0.7658	0.7724	0.136*	
C10	0.5424 (3)	0.8922 (6)	0.67926 (19)	0.1061 (10)	
H10	0.5059	1.0092	0.6955	0.127*	
C11	0.5502 (2)	0.8714 (4)	0.60893 (15)	0.0847 (8)	
H11	0.5206	0.9715	0.5775	0.102*	
C12	0.60455 (18)	0.6934 (4)	0.58821 (12)	0.0659 (6)	
C13	0.62725 (19)	0.6246 (4)	0.51808 (13)	0.0679 (6)	

### Atomic displacement parameters (Å^2^)

**Table:**

	*U*^11^	*U*^22^	*U*^33^	*U*^12^	*U*^13^	*U*^23^
Si1	0.0681 (4)	0.0500 (4)	0.0653 (4)	−0.0086 (3)	0.0081 (3)	−0.0030 (3)
O1	0.1094 (16)	0.0863 (13)	0.1218 (16)	0.0091 (12)	0.0198 (13)	0.0246 (12)
O2	0.0852 (13)	0.1100 (14)	0.0822 (13)	0.0171 (11)	−0.0001 (10)	0.0038 (10)
N1	0.0528 (11)	0.0727 (12)	0.0818 (13)	−0.0037 (9)	0.0134 (9)	−0.0067 (10)
C1	0.119 (2)	0.0547 (15)	0.125 (2)	−0.0060 (14)	0.0245 (19)	−0.0052 (15)
C2	0.0817 (19)	0.113 (2)	0.113 (2)	−0.0169 (17)	0.0352 (17)	−0.0301 (18)
C3	0.140 (3)	0.100 (2)	0.0790 (18)	−0.0160 (19)	−0.0120 (18)	0.0127 (15)
C4	0.0537 (13)	0.0573 (12)	0.0731 (14)	−0.0078 (10)	0.0040 (10)	−0.0068 (10)
C5	0.0557 (14)	0.0794 (16)	0.0971 (18)	−0.0096 (12)	0.0096 (13)	−0.0228 (13)
C6	0.0624 (15)	0.0761 (16)	0.0902 (18)	−0.0133 (13)	0.0135 (12)	0.0076 (14)
C7	0.0562 (13)	0.0845 (16)	0.0787 (16)	−0.0124 (12)	0.0180 (12)	0.0012 (13)
C8	0.088 (2)	0.129 (3)	0.0849 (19)	−0.0080 (18)	0.0235 (15)	0.0079 (18)
C9	0.091 (2)	0.167 (3)	0.086 (2)	−0.014 (2)	0.0268 (18)	−0.025 (2)
C10	0.078 (2)	0.124 (3)	0.120 (3)	−0.0098 (18)	0.0273 (19)	−0.045 (2)
C11	0.0559 (14)	0.0910 (18)	0.108 (2)	−0.0047 (13)	0.0119 (13)	−0.0201 (16)
C12	0.0427 (11)	0.0765 (15)	0.0791 (15)	−0.0121 (11)	0.0095 (10)	−0.0134 (13)
C13	0.0469 (12)	0.0777 (15)	0.0789 (16)	−0.0061 (11)	0.0040 (11)	−0.0031 (13)

### Geometric parameters (Å, °)

**Table:**

Si1—C1	1.862 (2)	C4—C5	1.522 (3)
Si1—C2	1.869 (3)	C4—H4A	0.9700
Si1—C4	1.869 (2)	C4—H4B	0.9700
Si1—C3	1.867 (3)	C5—H5A	0.9700
O1—C6	1.212 (3)	C5—H5B	0.9700
O2—C13	1.213 (3)	C6—C7	1.484 (4)
N1—C6	1.394 (3)	C7—C8	1.371 (4)
N1—C13	1.395 (3)	C7—C12	1.380 (3)
N1—C5	1.457 (3)	C8—C9	1.381 (5)
C1—H1A	0.9600	C8—H8	0.9300
C1—H1B	0.9600	C9—C10	1.367 (5)
C1—H1C	0.9600	C9—H9	0.9300
C2—H2A	0.9600	C10—C11	1.385 (4)
C2—H2B	0.9600	C10—H10	0.9300
C2—H2C	0.9600	C11—C12	1.380 (3)
C3—H3A	0.9600	C11—H11	0.9300
C3—H3B	0.9600	C12—C13	1.479 (3)
C3—H3C	0.9600		
			
C1—Si1—C2	110.31 (14)	H4A—C4—H4B	107.5
C1—Si1—C4	110.45 (11)	N1—C5—C4	113.77 (18)
C2—Si1—C4	107.87 (11)	N1—C5—H5A	108.8
C1—Si1—C3	109.30 (14)	C4—C5—H5A	108.8
C2—Si1—C3	110.16 (15)	N1—C5—H5B	108.8
C4—Si1—C3	108.73 (14)	C4—C5—H5B	108.8
C6—N1—C13	111.7 (2)	H5A—C5—H5B	107.7
C6—N1—C5	123.9 (2)	O1—C6—N1	124.2 (3)
C13—N1—C5	124.4 (2)	O1—C6—C7	130.1 (3)
Si1—C1—H1A	109.5	N1—C6—C7	105.8 (2)
Si1—C1—H1B	109.5	C8—C7—C12	121.1 (2)
H1A—C1—H1B	109.5	C8—C7—C6	130.6 (3)
Si1—C1—H1C	109.5	C12—C7—C6	108.3 (2)
H1A—C1—H1C	109.5	C7—C8—C9	117.3 (3)
H1B—C1—H1C	109.5	C7—C8—H8	121.3
Si1—C2—H2A	109.5	C9—C8—H8	121.3
Si1—C2—H2B	109.5	C10—C9—C8	121.4 (3)
H2A—C2—H2B	109.5	C10—C9—H9	119.3
Si1—C2—H2C	109.5	C8—C9—H9	119.3
H2A—C2—H2C	109.5	C9—C10—C11	122.1 (3)
H2B—C2—H2C	109.5	C9—C10—H10	119.0
Si1—C3—H3A	109.5	C11—C10—H10	119.0
Si1—C3—H3B	109.5	C10—C11—C12	116.1 (3)
H3A—C3—H3B	109.5	C10—C11—H11	122.0
Si1—C3—H3C	109.5	C12—C11—H11	122.0
H3A—C3—H3C	109.5	C11—C12—C7	122.1 (2)
H3B—C3—H3C	109.5	C11—C12—C13	129.7 (2)
C5—C4—Si1	115.09 (15)	C7—C12—C13	108.2 (2)
C5—C4—H4A	108.5	O2—C13—N1	124.5 (2)
Si1—C4—H4A	108.5	O2—C13—C12	129.5 (2)
C5—C4—H4B	108.5	N1—C13—C12	106.0 (2)
Si1—C4—H4B	108.5		
			
C1—Si1—C4—C5	−59.0 (2)	C8—C9—C10—C11	0.1 (5)
C2—Si1—C4—C5	−179.61 (18)	C9—C10—C11—C12	0.5 (4)
C3—Si1—C4—C5	60.9 (2)	C10—C11—C12—C7	−0.3 (4)
C6—N1—C5—C4	−80.8 (3)	C10—C11—C12—C13	−179.8 (2)
C13—N1—C5—C4	98.0 (3)	C8—C7—C12—C11	−0.4 (3)
Si1—C4—C5—N1	−174.99 (17)	C6—C7—C12—C11	−178.9 (2)
C13—N1—C6—O1	−177.9 (2)	C8—C7—C12—C13	179.2 (2)
C5—N1—C6—O1	1.0 (4)	C6—C7—C12—C13	0.7 (2)
C13—N1—C6—C7	1.7 (2)	C6—N1—C13—O2	179.6 (2)
C5—N1—C6—C7	−179.43 (18)	C5—N1—C13—O2	0.7 (3)
O1—C6—C7—C8	−0.2 (5)	C6—N1—C13—C12	−1.3 (2)
N1—C6—C7—C8	−179.7 (2)	C5—N1—C13—C12	179.83 (18)
O1—C6—C7—C12	178.1 (3)	C11—C12—C13—O2	−1.0 (4)
N1—C6—C7—C12	−1.4 (2)	C7—C12—C13—O2	179.4 (2)
C12—C7—C8—C9	1.0 (4)	C11—C12—C13—N1	179.9 (2)
C6—C7—C8—C9	179.1 (3)	C7—C12—C13—N1	0.3 (2)
C7—C8—C9—C10	−0.8 (5)		

### Hydrogen-bond geometry (Å, °)

**Table:**

*D*—H···*A*	*D*—H	H···*A*	*D*···*A*	*D*—H···*A*
C11—H11···O2^i^	0.93	2.57	3.443 (4)	156

Symmetry codes: (i) −*x*+1, −*y*+2, −*z*+1.
